# Determinants of left ventricular mass in children with autosomal recessive polycystic kidney disease

**DOI:** 10.1007/s40620-025-02426-y

**Published:** 2025-10-07

**Authors:** Mathew Lin, Jeremy Rubin, Robert A. Palermo, Jarcy Zee, Erum A. Hartung

**Affiliations:** 1 Division of Nephrology, Children’s Hospital of Philadelphia, Philadelphia, PA USA; 2 Department of Biostatistics, Epidemiology, and Informatics, Perelman School of Medicine at the University of Pennsylvania, Philadelphia, PA USA; 3 Division of Cardiology, Children’s Hospital of Philadelphia, Philadelphia, PA USA; 4 Department of Pediatrics, Perelman School of Medicine at the University of Pennsylvania, Philadelphia, PA USA; 5 Children’s Hospital of Philadelphia Research Institute, Philadelphia, PA USA

**Keywords:** Autosomal recessive polycystic kidney disease, Hypertension, Ambulatory blood pressure monitoring, Left ventricular mass, Left ventricular hypertrophy

## Abstract

**Background:**

Hypertension and left ventricular (LV) hypertrophy (LVH) are common in autosomal recessive polycystic kidney disease (ARPKD). We examined clinical determinants of LV mass in children with ARPKD.

**Methods:**

Retrospective study of patients with ARPKD with available echocardiogram data. Casual blood pressure (BP) percentiles, 24-h ambulatory BP monitor (ABPM) parameters, antihypertensive medications, and estimated glomerular filtration rate (eGFR) within 6 months of echocardiogram were collected. Outcomes included LV mass Z-score, LV mass index [LVMI in g/m^2.7^ and g/(m^2.16^ + 0.09)], and LVH.

**Results:**

Thirty patients with ARPKD (median age 7.2 years [IQR 3.4, 12.8]) had echocardiograms, 28 had casual BPs, 11 had ABPMs, and 93% were on antihypertensives. LVH occurred in 23% based on LVMI in g/m^2.7^ > 95th percentile, and in 50% based on LVMI > 45 g/(m^2.16^ + 0.09). Younger age correlated with higher number of antihypertensives (*ρ* = − 0.46, *P* = 0.014) and higher casual systolic and diastolic BP percentiles (*r* = − 0.74, *P* < 0.001; *r* = − 0.81, *P* < 0.001). After adjusting for age, sex, and eGFR, LV mass was not significantly associated with casual BP or ABPM, except for a negative association between LV mass Z-score and casual diastolic BP percentile (β coefficient − 0.31, *P* = 0.04). After adjusting for age, sex, and casual BP, both LVMI [in g/m^2.7^ and g/(m^2.16^ + 0.09)] and LV mass Z-score were significantly negatively associated with eGFR (β –1.08, *P* = 0.003; β –0.79, *P* = 0.007; and β -0.07, *P* = 0.01, respectively).

**Conclusions:**

Young children with ARPKD have a higher burden of hypertension. LV mass was unexpectedly not significantly associated with BP but was negatively associated with eGFR.

**Graphical abstract:**

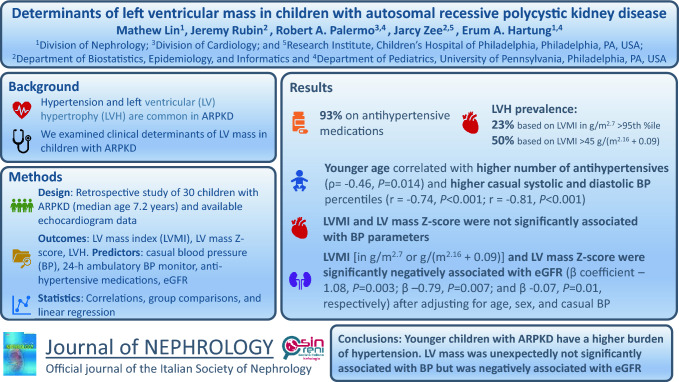

**Supplementary Information:**

The online version contains supplementary material available at 10.1007/s40620-025-02426-y.

## Introduction

Autosomal recessive polycystic kidney disease (ARPKD) is a rare genetic hepatorenal fibrocystic disease with an estimated incidence of 1:26,500 live births [1]. The vast majority of ARPKD is caused by pathogenic variants in the *PKHD1* gene encoding fibrocystin/polyductin, a protein expressed in the epithelia of kidney tubules and bile ducts as well as in the muscular walls of large blood vessels [2, 3]. The clinical spectrum of ARPKD is highly variable. A subset of infants presents in the perinatal period with massively enlarged kidneys, oligohydramnios, and pulmonary hypoplasia, with a high risk of early dialysis dependency and mortality [4–6]. However, severe perinatal presentation accounts for fewer than half of patients, and many are diagnosed later in infancy, childhood, adolescence, or rarely even in adulthood [4, 5, 7]. ARPKD is characterized by enlarged kidneys with numerous microcysts arising from dilated collecting ducts, with progressive chronic kidney disease (CKD) leading to chronic kidney failure at varying ages [8]. Arterial hypertension is a very common comorbidity in patients with ARPKD, occurring in about 85% of patients [8–11]. Hypertension often presents very early in life and precedes a significant decline in kidney function [12]. In one cohort study, hypertension was diagnosed at birth in 38% of patients, between 0–1 year of age in 31% of patients, and between 1 and 6 years of age in 25% of patients [7, 8]. Hypertension is often severe and may require multi-drug therapy [9, 11], particularly in infancy and early childhood [7, 10].

Given this high burden of hypertension in children with ARPKD, abnormalities in cardiac geometry and function are also quite prevalent. Left ventricular hypertrophy (LVH) has been reported in 20–33% of children with ARPKD in various cohort studies [9, 10, 13]. Chinali et al. [11] reported higher left ventricular mass index (LVMI) in a cohort of 27 children with ARPKD compared to healthy controls, which was independently associated with higher systolic blood pressure (SBP) Z-score but not with diastolic blood pressure (DBP) or estimated glomerular filtration rate (eGFR). In a retrospective study of 36 children with ARPKD who had undergone ambulatory blood pressure monitoring (ABPM), Seeman et al. [10] found LVH in 27% of children at time of their first ABPM; 94% of those children had ambulatory hypertension, but determinants of left ventricular (LV) mass were not specifically evaluated. Dell et al. [9] found LVH in 20% of the 22 children evaluated in the Chronic Kidney Disease in Children (CKiD) cohort study; again, there was a high prevalence of hypertension in this cohort (86% of children were receiving antihypertensive therapy), but determinants of LV mass were not explored.

Although LVH in children with ARPKD is presumed to arise from the high burden of hypertension, there are gaps in knowledge regarding the impact of other risk factors such as age, sex, kidney function, and antihypertensive medications. Also, since fibrocystin/polyductin is expressed in vascular walls [2], it is possible that ARPKD may have a primary effect on cardiovascular health independent of hypertension. The objective of this study was to examine determinants of LV mass in children with ARPKD, including casual and 24-h ambulatory BP measures, antihypertensive medication use, age, sex, and kidney function.

## Methods

### Participants

This is a single-center retrospective study of patients with ARPKD who received care at the Children’s Hospital of Philadelphia (CHOP). The study was determined by the CHOP Institutional Review Board (IRB 21–018989) to meet exemption criteria per Title 45 of the U.S. Code of Federal Regulations §46.104(d) 4(iii) and a waiver of HIPAA authorization/consent was granted. Eligible participants had a clinical diagnosis of ARPKD and available echocardiogram data in the electronic health record (EHR) prior to kidney transplantation or dialysis. If patients had multiple echocardiograms, only data pertaining to the most recent echocardiogram were included in this study. Diagnosis of ARPKD was made by the treating physician based on clinical criteria, including enlarged, echogenic kidneys, decreased cortico-medullary differentiation, coexisting liver disease (e.g., heterogeneous and/or increased liver echotexture), and family history consistent with autosomal recessive inheritance [14]. Genetic testing results were recorded if available, but genetic confirmation of an ARPKD diagnosis was not required for inclusion in this study. All data after kidney transplantation or dialysis were excluded from the study.

### Echocardiographic data

Patients underwent transthoracic echocardiograms according to standard clinical protocols using commercially available machines. LV mass was calculated using the Devereux formula [15, 16] from M-mode measurements at end-diastole of interventricular septal thickness, posterior wall thickness, and LV diameter. LV mass Z-score was calculated using the mass-for-height percentile method described by Foster et al. [17]. LVMI was calculated using two methods, by indexing LV mass to: (1) height to the power of 2.7 (g/m^2.7^) [18], and (2) height to the power of 2.16 with a correction factor of 0.09 [g/(m^2.16^ + 0.09)] [19]. LVH was defined as LVMI > 95th percentile for age based on reference intervals from Khoury et al. [20] for LVMI in g/m^2.7^, or as LVMI > 45 g/(m^2.16^ + 0.09) as defined by Chinali et al. [19]. For patients without sufficient M-mode measurements in the echocardiogram report to calculate LV mass, LVH was classified based on the cardiologist’s qualitative assessment in the report narrative.

### Clinical data

Available clinical data including anthropometrics, medications, laboratory data, casual blood pressures, and 24-h ABPM were collected from the EHR from clinical visits closest in time to the echocardiogram, up to a maximum of 6 months before or after the echocardiogram.

#### eGFR

eGFR was calculated based on serum creatinine using the CKiD U25 equation [21]. Due to the retrospective nature of the study, serum cystatin C was not available in most patients.

#### Antihypertensive medications

Antihypertensive medications were recorded from the nephrology clinic visit closest to the echocardiogram. Medications were classified into ACE inhibitors (ACE-Is), angiotensin II receptor blockers (ARBs), beta blockers (BBs), calcium channel blockers (CCBs), and centrally acting alpha-2 agonists (CAAAs).

#### Casual blood pressures

Casual BP readings were collected from the day of the echocardiogram and up to three additional nephrology clinic visits within 6 months of the echocardiogram. The first documented BP reading from each encounter was recorded. SBP and DBP percentiles for each casual BP reading were calculated based on normative data for age, sex, and height [22]. An overall mean casual SBP and DBP percentile for each patient was generated from all available readings (up to 4 readings per patient). The severity of hypertension was staged by a modified hypertension score [23, 24], with 1 point for each antihypertensive agent (based on medications at the visit closest to the echocardiogram) and 1 point for presence of hypertension, defined as overall mean casual SBP and/or DBP percentile > 95th percentile.

#### ABPM Measurements

24-h ABPM was performed using oscillometric SpaceLabs monitors (SpaceLabs Healthcare, Snoqualmie, WA, USA). Variables collected from the ABPM included mean SBP and DBP values during the overall 24-h period, wake, and sleep. Percent dipping between wake and sleep values was recorded. To allow comparison of ABPM data across age groups, we generated 4 indexed variables: wake SBP index, wake DBP index, sleep SBP index, and sleep DBP index. Indexes were calculated by dividing the mean SBP or DBP during wake or sleep by the upper limit of normal for age and sex, as defined by 2022 American Heart Association guidelines (for ≥ 13 years of age: 130/80 mmHg wake and 110/65 sleep; for < 13 years of age: lower value of 95th percentile or adolescent cut points) [25].

#### Analysis

Pearson’s correlation was used to examine relationships between age, eGFR, casual BP percentiles, ABPM BP indexes, LVMI, and LV mass Z-score. Spearman’s correlation was used to examine relationships between the number of antihypertensive medications, hypertension score, and other variables. For all variables except LV mass Z-score, we computed medians and interquartile ranges (IQRs) for subjects stratified by LVH status as well as the *P*-value from a two-sample Wilcoxon rank sum test. For LV mass Z-score, we computed means and standard deviations for subjects stratified by sex and a *P*-value from a two-sample t-test.

Linear regression was used to assess relationships between LVMI and LV mass Z-score, ABPM indexes, BP, and eGFR, with and without adjusting for age and sex. LVMI and LV mass Z-score were the primary outcomes of interest, while ABPM indexes, eGFR and casual SBP and DBP percentile were the primary predictors in the analysis.

## Results

### Patient characteristics

We identified 30 eligible patients with ARPKD and available echocardiogram data from 2000 to 2022, 11 of whom had ABPM data. Genetic testing results were available in 20 patients, all of whom had at least one *PKHD1* variant detected. Of these, five patients had homozygous *PKHD1* variants, and 13 patients had compound heterozygous *PKHD1* variants. Three patients who had not undergone genetic testing had a full sibling with a genetically confirmed ARPKD diagnosis. Thus, 23 of 30 patients (77%) had genetic evidence of ARPKD. In the full cohort, the median age at the time of echocardiogram was 7.2 (range 0.2–19.4) years, with a median eGFR of 49 mL/min/1.73 m^2^. In the ABPM cohort, median age at the time of echocardiogram was 11.5 years, with a median eGFR of 45 mL/min/1.73m^2^ (Table 1).

**Table 1:** Clinical and demographic characteristics

Variable	Full cohort (*n* = 30)		ABPM cohort (*n* = 11)	
	n^1^	Value	n^1^	Value
**Clinical and demographic characteristics**				
Female sex	30	13 (43%)	11	4 (36%)
eGFR (mL/min/1.73m^2^)	30	49 (23, 75)	11	45 (36, 64)
**Echocardiogram**				
Age at echocardiogram (years)	30	7.2 (3.4, 12.8)	11	11.5 (8,2, 14.8)
LV mass (g)	28	64.8 (43.5, 104.3)	10	121.8 (73.0, 157.7)
LVMI (g/m^2.7^)	28	40.5 (33.5, 67.8)	10	37.0 (31.2, 43.8)
LVH present (Khoury criteria)^3^	30	7 (23%)	11	2 (18%)
LVMI (g/m^2.16^ + 0.09)	28	47.2 (34.1, 54.4)	10	44.8 (36.7, 57.3)
LVH present (Chinali criteria)^4^	30	15 (50%)	11	5 (45%)
LV mass Z-score	28	0.54 (−0.92, 1.22)	10	0.14 (−0.54, 1.13)
**Clinic blood pressure**				
Number of antihypertensive medications^5^	28		11	
0		2 (7%)		0 (0%)
1		15 (54%)		8 (73%)
2		6 (21%)		2 (18%)
3		5 (18%)		1 (9%)
Treated patients with uncontrolled hypertension^6^	26	5 (19%)	11	1 (9%)
Mean casual SBP percentile^7^	28	77 (63, 82)	11	64 (51, 78)
Mean casual DBP percentile^7^	28	71 (45, 85)	11	40 (19, 66)
Hypertension score^8^	28	1 (1, 2.25)	11	1 (1, 2)
**ABPM**				
Age at ABPM (years)			11	11.5 (8.7, 14.9
Overall average SBP (mmHg)			11	107 (98, 120)
Overall average DBP (mmHg)			11	66 (60, 74)
Wake SBP index^9^			11	0.93 (0.84, 0.98)
Wake DBP index^9^			11	0.90 (0.82, 0.96)
Sleep SBP index^9^			11	0.92 (0.87, 1.01)
Sleep DBP index^9^			11	0.92 (0.87, 0.98)
Nocturnal SBP dipping (%)			11	10.5 (4.7, 15.0)
Nocturnal DBP dipping (%)			11	14.6 (12.1, 19.2)

Continuous variables are expressed as median (interquartile range). Proportions are expressed as number (percentage)

*ABPM* 24-h ambulatory blood pressure monitor, *DBP* diastolic blood pressure, *eGFR* estimated glomerular filtration rate, *LVH* left ventricular hypertrophy, *LVMI* left ventricular mass index, *LV mass Z-score* left ventricular mass normalized to LV mass-for-height Z-score, *MAP* mean arterial pressure, *SBP* systolic blood pressure

^1^Number of subjects with available data for variable

^2^Calculated using CKiD U25 creatinine-based equation [21], https://ckid-gfrcalculator.shinyapps.io/eGFR/

^3^LVH defined as LVMI > 95th percentile for age based on Khoury, PR et al. [20] and/or qualitative LVH noted in report

^4^LVH defined as LVMI > 45 (g/m^2.16^ + 0.09) based on Chinali et al. [19] and/or qualitative LVH noted in report

^5^Mean number of BP meds across up to 3 clinic visits within 6 months of echocardiogram

^6^Proportion of patients on antihypertensive therapy with mean casual SBP or DBP > 95th percentile

^7^Mean casual SBP and DBP percentile = mean percentile of casual SBP and DBP on day of echocardiogram and casual BPs recorded at up to 3 clinic visits within 6 months of echocardiogram

^8^Hypertension score: 1 point for each antihypertensive agent (based on medications at the visit closest to the echocardiogram) and 1 point for presence of hypertension, defined as overall mean casual SBP and/or DBP percentile > 95th percentile

^9^ABPM BP indexes were calculated by dividing the mean SBP or DBP during wake or sleep by the upper limit of normal for age and sex, as defined by 2022 American Heart Association guidelines (for ≥ 13 years of age: 130/80 mmHg wake and 110/65 sleep; for < 13 years of age: lower value of 95th percentile or adolescent cut points) [25]

### Blood pressures and antihypertensive therapy

In the full cohort, median casual SBP was at the 77th percentile, median casual DBP was at the 71st percentile, and median hypertension score was 1 (Table 1). Casual SBP was positively correlated with casual DBP (*r* = 0.80, *P* < 0.001; Supplementary Table 1). In the ABPM cohort, median casual SBP was at the 64th percentile and median casual DBP was at the 40th percentile; median SBP and DBP indexes during wake and sleep ranged from 0.90 to 0.93, as shown in Table 1. ABPM SBP indexes during wake and sleep were positively correlated (*r* = 0.70, *P* = 0.02); similarly, DBP indexes during wake and sleep were positively correlated (*r* = 0.71, *P* = 0.02; Supplementary Table 1). ABPM SBP index was not significantly correlated with DBP index during wake or sleep (Supplementary Table 1). ABPM wake DBP index was positively correlated with casual DBP percentile (*r* = 0.71, *P* = 0.02) but there were no other significant correlations between ABPM SBP and DBP indexes and casual BPs. Hypertension score was positively correlated with ABPM sleep DBP index (*r* = 0.76, *P* = 0.006) but was not significantly correlated with any other ABPM indexes (Supplementary Table 1).

**Fig. 1:**
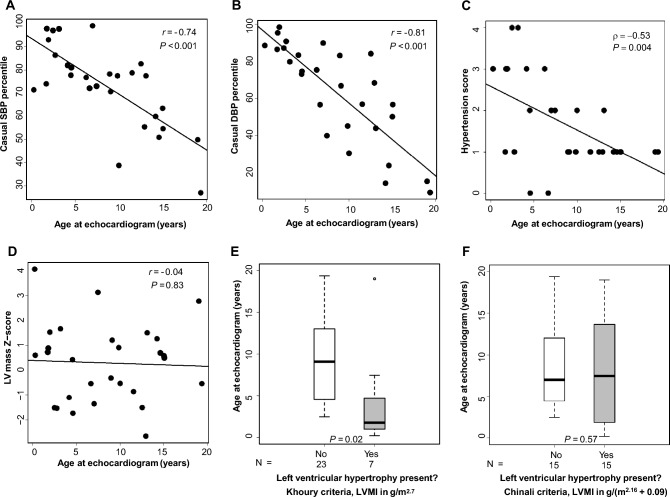
Relationship between age, casual blood pressure (BP), hypertension score, and left ventricular geometry in patients with autosomal recessive polycystic kidney disease (ARPKD).** A** Scatterplot of age at echocardiogram and casual systolic BP (SBP) percentile; **B** Scatterplot of age at echocardiogram and casual diastolic BP (DBP) percentile; **C** Scatterplot of age at echocardiogram and hypertension score; **D** Scatterplot of age at echocardiogram and left ventricular (LV) mass Z-score; **E** Box plot of age at echocardiogram stratified by presence of left ventricular hypertrophy (Khoury et al. criteria, [20]); **F.** Box plot of age at echocardiogram stratified by presence of left ventricular hypertrophy (Chinali et al. criteria, [19])

Casual SBP and DBP percentiles were both significantly negatively correlated with age (*r* = − 0.74, *P* < 0.001, and *r* = − 0.81, *P* < 0.001, respectively; Supplementary Table 1 and Fig. 1A, B). ABPM wake DBP index was negatively correlated with age (*r* = − 0.61, *P* = 0.048), but remaining ABPM indexes were not significantly correlated with age (Supplementary Table 1). Neither casual BP percentiles nor ABPM indexes were significantly correlated with eGFR (Supplementary Table 1).

Most patients [93% (26/28; missing medication data in *n* = 2)] were on antihypertensive medications. Combinations of classes of antihypertensive therapy used in patients at the visit closest to the echocardiogram are shown in Supplementary Fig. 1. ACE-I was the most common class of medications used (*n* = 17 patients total, as monotherapy in *n* = 12 patients). The number of antihypertensive medications was negatively correlated with age at echocardiogram (ρ = − 0.46, *P* = 0.014) and positively correlated with eGFR (*ρ* = 0.43, *P* = 0.022) but was not significantly correlated with casual SBP or DBP percentiles (*ρ* = 0.28, *P* = 0.15 and ρ = 0.30, *P* = 0.11, respectively) or ABPM SBP or DBP indexes (ranges of *ρ* = −0.31 to 0.41, *P* = 0.21 to 0.49). Five of the 26 patients receiving antihypertensive therapy (19%) had uncontrolled hypertension (i.e. SBP or DBP > 95th percentile (Table 1)). Hypertension score was negatively correlated with age (ρ = −0.53, *P* = 0.004, Fig. 1C); there was a trend towards negative correlation with eGFR, but this did not reach statistical significance (*ρ* = −0.37, *P* = 0.06; Supplementary Table 1).

### Determinants of LV mass

In the full cohort, 23% of patients (7 of 30) had LVH by Khoury et al. criteria [20], with median LVMI of 40.5 g/m^2.7^. Using Chinali et al. criteria [19], 50% of patients (15 of 30) had LVH, with median LVMI of 47.2 g/(m^2.16^ + 0.09). Median LV mass Z-score was 0.54 (Table 1).

#### Age and sex

LVMI in g/m^2.7^ was negatively correlated with age (*r* = −0.48, *P* = 0.01), while LVMI in g/(m^2.16^ + 0.09) was not significantly correlated with age (*r* = −0.033, *P* = 0.87), consistent with observations in healthy reference populations [19, 20](Supplementary Table 1). LV mass Z-score was not significantly correlated with age (*r* = −0.04, P = 0.83, Fig. 1D). Children who had LVH by Khoury et al. criteria [20] were significantly younger than those without LVH (median 1.76 vs. 9.08 years, *P* = 0.02, Fig. 1E), but there was no significant group difference in median age by LVH status when LVH was classified by Chinali et al. criteria [19](Fig. 1F). LV mass Z-score was significantly higher in males than in females (1.03 ± 1.63 vs. −0.54 ± 1.06, *P* = 0.005).

#### Blood pressure

**Fig. 2:**
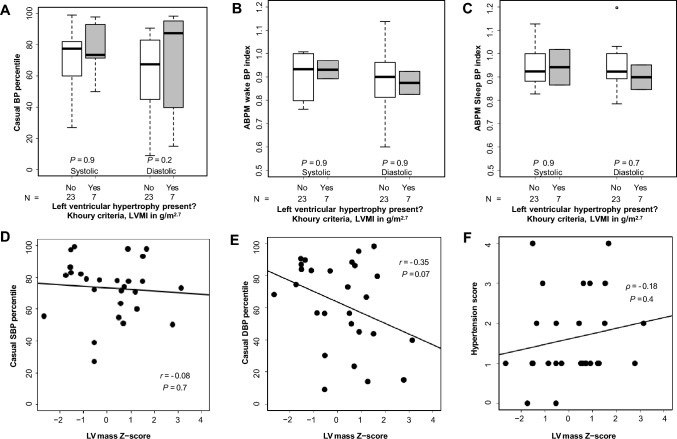
Relationship between left ventricular hypertrophy (LVH) by Khoury et al. criteria [20], left ventricular (LV) mass Z-score, and blood pressure (BP) in patients with autosomal recessive polycystic kidney disease (ARPKD). **A** Box plot of casual systolic BP (SBP) and diastolic BP (DBP) percentiles in patients with and without LVH; **B** Box plot of ambulatory blood pressure monitor (ABPM) wake SBP and DBP indexes in patients with and without LVH; **C** Box plot of ABPM sleep SBP and DBP indexes in patients with and without LVH; **D** Scatterplot of casual SBP percentile and LV mass Z-score; **E** Scatterplot of casual DBP percentile and LV mass Z-score. **F** Scatterplot of hypertension score and LV mass Z-score

Comparisons of patients with and without LVH by Khoury et al. criteria [20] showed no significant differences in overall mean casual SBP and DBP percentiles (SBP percentile 73.5 vs. 77.5, *P* = 0.9; DBP percentile 87.4 vs. 67.5, *P* = 0.2, Fig. 2A). ABPM parameters were also not significantly different between patients with and without LVH (wake SBP index 0.93 vs. 0.93, *P* = 0.9; wake DBP index 0.87 vs. 0.90, *P* = 0.9; sleep SBP index 0.94 vs. 0.92, *P* > 0.9; sleep DBP index 0.90 vs. 0.92, *P* = 0.7; Fig. 2B, C). Comparisons of patients with and without LVH by Chinali et al. criteria [19] similarly showed no significant differences in overall mean casual BP percentiles or ABPM indexes (Supplementary Fig. 2).

There were no significant linear correlations between LV mass Z-score and casual SBP or DBP percentiles (*r* = −0.08, *P* = 0.7; and *r* = −0.35, *P* = 0.07, respectively; Figs. 2D, E). The number of antihypertensive medications was positively correlated with LVMI in g/m^2.7^ (*ρ* = 0.55, *P* = 0.003), but was not significantly correlated with LVMI in g/(m^2.16^ + 0.09) (*ρ* = 0.32, *P* = 0.10) or LV mass Z-score (*ρ* = −0.29, *P* = 0.2). Hypertension score was positively correlated with LVMI in g/m^2.7^ (*ρ* = 0.52, *P* = 0.005) but was not significantly correlated with LVMI in g/(m^2.16^ + 0.09) (*ρ* = 0.16, *P* = 0.4) or LV mass Z-score (*ρ* = 0.18, *P* = 0.4) (Supplementary Table 1).

In univariate linear regression analysis, LVMI in g/m^2.7^ was positively associated with hypertension score and LVMI in g/(m^2.16^ + 0.09) was negatively associated with mean casual DBP percentile. There were otherwise no significant associations between LVMI [in either g/m^2.7^ or g/(m^2.16^ + 0.09)] or LV mass Z-score and any other BP predictor variables, including casual SBP and DBP percentiles and ABPM BP indexes (Supplementary Table 2).

After adjustment for age, sex, and eGFR, linear regression analysis showed that LV mass Z-score was negatively associated with casual DBP percentile (β coefficient −0.31, *P* = 0.04) but there were otherwise no significant associations between LV geometry outcomes and any BP predictor variables (Supplementary Table 2).

#### eGFR

There were no significant linear correlations between eGFR and age, casual BPs, ABPM BP indexes (Supplementary Table 1).

**Fig. 3:**
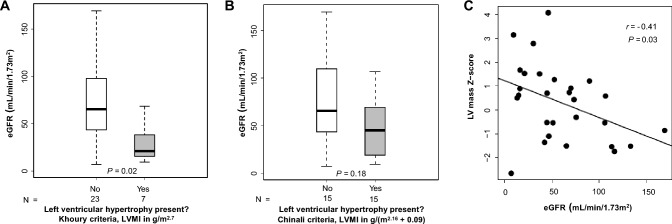
Relationship between left ventricular geometry and estimated glomerular filtration rate (eGFR). A**.** Box plot of eGFR stratified by left ventricular hypertrophy (LVH) status (Khoury et al. criteria, [20]); **B** Box plot of eGFR stratified by presence of LVH (Chinali et al. criteria, [19]); **C** Scatterplot of eGFR by LV mass Z-score

Patients with LVH by Khoury et al. criteria [20] had a significantly lower eGFR than those without LVH (21 vs*.* 65 mL/min/1.73 m^2^, *P* = 0.02, Fig. 3A) but there was no significant difference in eGFR in patients with versus without LVH by Chinali et al. criteria [20] (45 vs*.* 65 mL/min/1.73m^2^, *P* = 0.18, Fig. 3B). Significant negative correlations were found between eGFR and LVMI in g/(m^2.16^ + 0.09) (*r* = −0.43, *P* = 0.02, Supplementary Table 1) and between eGFR and LV mass Z-score (*r* = −0.41, *P* = 0.03, Fig. 3C). In unadjusted univariate linear regression, both LVMI in g/(m^2.16^ + 0.09) and LV mass Z-score were negatively associated with eGFR (β coefficient −0.84, *P* = 0.02 and β coefficient −0.07, *P* = 0.03, respectively; Supplementary Table 2). After adjusting for age and sex, both LVMI [in g/m^2.7^ and g/(m^2.16^ + 0.09)] and LV mass Z-score were found to be negatively associated with eGFR (β coefficient −1.45, *P* = 0.03; β coefficient −0.82, *P* = 0.01; and β coefficient −0.08, *P* = 0.02, respectively; Supplementary Table 2). After further adjustment for mean casual SBP and DBP percentiles, in addition to age and sex, there were still significant negative associations between both LVMI [in g/m^2.7^ and g/(m^2.16^ + 0.09)] and LV mass Z-score and eGFR (β coefficient –1.08, *P* = 0.003; β coefficient –0.79, *P* = 0.007; and β coefficient −0.07, *P* = 0.01, respectively; Supplementary Table 2). Similar negative associations were found between both LVMI and LV mass Z-score and eGFR after adjustment for age, sex, and hypertension score (Supplementary Table 2).

## Discussion

The objective of this study was to investigate the determinants of LV mass in children with ARPKD, including BP from both casual and ABPM measurements and clinical variables such as antihypertensive medication use, age, sex, and kidney function.

Contrary to our expectations, LV mass was not significantly associated with casual BP percentiles, ABPM BP indexes, or hypertension score in this cohort of children with ARPKD after adjusting for age and sex, other than an unexpected negative association between LV mass Z-score and casual diastolic BP. We found that children with LVH defined by Khoury et al. criteria (LVMI in g/m^2.7^) [20] were significantly younger than those without LVH [19, 20], but there was no significant age difference by LVH status when defined by Chinali et al. criteria [LVMI in g/(m^2.16^ + 0.09)] [19]. In our cohort, more children were classified as having LVH using Chinali et al. criteria (15/30, 50%) compared to Khoury et al. criteria (7/30, 23%). The reason for this difference is unclear but may arise from differences in demographic characteristics and/or age distribution of the reference populations in the respective studies. The optimal echocardiographic definition of LVH remains unclear in the pediatric population. The American Academy of Pediatrics uses an LVMI of > 51 g/m^2.7^ to define LVH, but this threshold is recommended only for children > 8 years due the known increase in LVMI in g/m^2.7^ in younger and smaller children (< 9 years old and < 140 cm height) [22]. However, we used age- and sex-specific reference values for 95th percentile derived by Khoury et al. to define LVH across all ages [20], which should have overcome the known variations in younger children. The newer indexing method proposed by Chinali et al. for LVMI in g/(m^2.16^ + 0.09) does not vary significantly by age and height, and was therefore recommended by the authors to use in the smaller, younger population. This method also allows the use of a simplified single partition value of > 45 g/(m^2.16^ + 0.09) across the pediatric age spectrum [19]. Interestingly, in our population, the Chinali method resulted in a higher number of older children being classified as having LVH. Given the ongoing uncertainty in the field on the best way to define LVH in children, we chose to report results from both indexing methods to allow further comparisons in future studies.

We found that younger age was significantly associated with higher casual BPs, greater need for antihypertensive therapy, and higher hypertension score. Given the discrepancy in LVH classification between the Khoury et al. [20] and Chinali et al. [19] methods as described above, it is difficult to ascertain whether difficult-to-control hypertension in infants and young children with ARPKD contributes to LVH [20]. However, our observations that BP measurements and hypertension score were not independently associated with LV mass in all our other analyses suggests that hypertension is not the primary driver of LVH in this population.

We found that decreased kidney function was the primary determinant of increased LV mass in this cohort. Patients with LVH (by Khoury et al. criteria[20]) had significantly lower eGFR than patients without LVH, and in regression analyses adjusted for age, sex, and BP (either as casual BP percentiles or hypertension score), eGFR was independently negatively associated with LVMI by both indexing methods [19, 20] and with LV mass Z-score.

Our findings are consistent with prior studies in patients with ARPKD in several ways. First, our cohort had a very high prevalence of hypertension, with 93% of patients receiving antihypertensive therapy. Prior studies have reported hypertension (defined as antihypertensive medication use and/or casual BP or ABPM readings > 95th percentile) in 65–86% of children with ARPKD [8–11]. The prevalence of hypertension in our cohort was likely particularly high because patients were only included if they had undergone echocardiography, which is most often performed for a clinical indication of hypertension. Many patients in our cohort required multiple antihypertensive medications, with 6 of 28 patients (21%) requiring two medications and 5 of 28 patients (18%) requiring three medications. Similarly, Chinali et al. [11] reported 5 of 27 patients (19%) in their cohort required three antihypertensive medications, and Dell et al. [9] reported 7 of 22 participants (32%) in the CKiD study were on at least three antihypertensive medications. The prevalence of LVH in our cohort was 23% when defined by Khoury et al. criteria [20], which is similar to the prevalence of 20–33% reported in previous cohort studies [9, 10, 13], but the prevalence of LVH in our cohort was notably higher (50%) when defined by Chinali et al. criteria [19].

The lack of association in our cohort between LV mass and BP variables contrasts with findings from the only previous study to directly examine determinants of LV mass in children with ARPKD. In a cohort of 27 children with ARPKD, Chinali et al. found that LVMI [in g/(m^2.16^ + 0.09)] was independently associated with casual systolic BP Z-score [11]. Our findings also contrast with studies in other populations. Studies in both children and adults with autosomal *dominant* polycystic kidney disease (ADPKD) have found positive associations between LV mass and BP [26, 27]. Extensive literature in children and adults with other causes of CKD and in the general population also documents associations between higher LV mass and higher BP [28, 29]. However, some prior studies have suggested a weaker link between LV mass and BP. For example, in a study of 126 adults with ADPKD, Chen et al. [30] found no difference in the prevalence of LVH between patients with and without hypertension. In adolescents without chronic medical conditions, casual SBP percentile alone was a weak (R^2^ = 0.11) but statistically significant predictor of LVMI [31]. It is therefore possible that the relatively small size of our cohort did not allow us to detect a weak association between BP and LV mass.

In our cohort, the primary independent determinant of increased LV mass was decreased eGFR. This finding contrasts with the study of 27 children with ARPKD by Chinali et al. [11], which did not show eGFR to be an independent predictor of LVMI. However, that study did find an association between eGFR and midwall fractional shortening that was independent of BP, suggesting a relationship between kidney function and cardiac dysfunction in children with ARPKD [11]. Studies in adults with ADPKD have also not shown a clear relationship between LV mass and kidney function. Chen et al. [30] found no independent relationship between eGFR and LV mass in a cohort of 126 adults with ADPKD (although they did find a relationship between LV mass and total kidney volume, suggesting some effect of ADPKD disease severity on LV mass). Similarly, in a study of 543 hypertensive adults with ADPKD who underwent cardiac magnetic resonance imaging, Perrone et al. [32] also reported no association between LV mass and eGFR. However, each of these ADPKD cohorts included patients with relatively preserved kidney function (median eGFR 63 and 92.5 mL/min/1.73 m^2^, respectively [30, 32]) which may have affected their ability to detect the effects of more advanced CKD on LV mass. Although these prior studies in ARPKD and ADPKD did not document relationships between LV mass and eGFR, studies in other CKD populations have shown a direct relationship between kidney function and cardiac remodeling, independent of BP. Uremic cardiomyopathy, a well-described phenomenon in children and adults with advanced CKD, generally manifests as LVH and diastolic dysfunction and is a multifactorial process [33]. Contributors to LVH, aside from hypertension in individuals with CKD, include CKD mineral and bone disorder (e.g., fibroblast growth factor 23, hyperparathyroidism), anemia, uremic toxins, and inflammation [33]. Thus, our finding that lower eGFR is independently associated with higher LV mass in this cohort of children with ARPKD is consistent with literature in other CKD populations.

Given the expression of fibrocystin/polyductin in vascular walls [2], there is also a possibility that ARPKD causes direct cardiovascular effects that are independent of BP and eGFR. However, our study cannot adequately answer this question since our cohort had a very high prevalence of hypertension and decreased eGFR.

Our study is limited primarily by small sample size and its retrospective nature. While our cohort size is comparable to other studies carried out on ARPKD pediatric populations, the relative rarity of the disease makes it difficult to draw general conclusions. Additionally, due to the nature of our study design, casual BPs were not collected in a standardized manner and were reliant on the values entered into the EHR during each patient’s clinic and echocardiogram visits. The technical difficulties of obtaining accurate BP readings in infants in real-world settings adds additional uncertainty regarding BP data in that subgroup. Since our analyses used BP readings closest to the time of echocardiogram, we cannot determine the impact of historic BP control on LV mass in our cohort. The retrospective nature of our study also means that we were unable to assess advanced echocardiographic parameters such as global longitudinal strain, since these measures were not part of standard clinical protocols at our center. Our ongoing prospective study in children with ARPKD, which includes advanced echocardiography, ABPM, and other comprehensive measures of cardiovascular function, will help to further characterize cardiovascular health in this population.

## Conclusion

In this retrospective study of 30 children with ARPKD, we found a high prevalence of hypertension and LVH in children, with a higher burden of hypertension in younger children. LV mass was not significantly associated with casual BP percentiles or 24-h ABPM BP indexes, but we did identify an independent association between lower eGFR and higher LV mass in children with ARPKD. Our study emphasizes the need for close attention to cardiovascular health in children with ARPKD, particularly in infants and in patients with advanced CKD.

## Supplementary Information

Below is the link to the electronic supplementary material.

Supplementary file1 (DOCX 291 KB)

Supplementary file2 (PDF 63 KB)

Supplementary file3 (PDF 125 KB)

## Data Availability

The datasets generated and analyzed during the current study are available from the corresponding author on reasonable request.
