# Supplementary material for: Determinants of left ventricular mass in children with autosomal recessive polycystic kidney disease
Source: J Nephrol. Author manuscript; Available in PMC 2025 Dec 22. (PMC12712119; doi:10.1007/s40620-025-02426-y)
Supplement: Supplementary File 1 [file NIHMS2124282-supplement-Supplementary_File_1.docx]

Determinants of left ventricular mass in children with autosomal recessive polycystic kidney disease

Mathew Lin, Jeremy Rubin, Robert A. Palermo, Jarcy Zee, Erum A. Hartung

# Supplementary information

# Supplementary Tables

## Supplementary Table 1

**Correlation matrix to show relationships between age, eGFR, casual BP percentiles, ABPM BP indexes, LVMI, and LV mass Z-score in patients with ARPKD.**

| Pearson’s r or Spearman’s ρ^*^  *P value* | Age | Casual SBP %ile | Casual DBP %ile | Wake SBP index | Wake DBP index | Sleep SBP index | Sleep DBP index | Hyper-tension score | LVMI (g/m^2.7^) | LVMI  (g/ m^2.16^  +0.09) | LV mass Z-score | eGFR |
| --- | --- | --- | --- | --- | --- | --- | --- | --- | --- | --- | --- | --- |
| Age^1^ | - |  |  |  |  |  |  |  |  |  |  |  |
| Casual SBP %ile^2^ | **-0.74**  ***<0.001*** | - |  |  |  |  |  |  |  |  |  |  |
| Casual DBP %ile^2^ | **-0.81 *<0.001*** | **0.80**  ***<0.001*** | - |  |  |  |  |  |  |  |  |  |
| Wake SBP index^3^ | -0.15  *0.66* | 0.54  *0.09* | 0.34  *0.30* | - |  |  |  |  |  |  |  |  |
| Wake DBP index^3^ | **-0.61**  ***0.048*** | 0.57  *0.07* | **0.71**  ***0.02*** | 0.58  *0.06* | - |  |  |  |  |  |  |  |
| Sleep SBP index^3^ | 0.21  *0.53* | 0.21  *0.54* | -0.08  *0.81* | **0.70**  ***0.02*** | 0.17  *0.62* | - |  |  |  |  |  |  |
| Sleep DBP index^3^ | -0.55  *0.08* | 0.39  *0.24* | 0.49  *0.12* | 0.17  *0.62* | **0.71**  ***0.02*** | 0.26  *0.43* | - |  |  |  |  |  |
| Hypertension score | **-0.53 *0.004*** | **0.48**  ***0.01*** | **0.42**  ***0.03*** | -0.15 0.66 | 0.42 0.20 | -0.15 0.65 | **0.76 *0.006*** | - |  |  |  |  |
| LVMI  (g/m^2.7^)^4^ | **-0.48**  ***0.01*** | 0.25  *0.21* | 0.17  *0.40* | 0.21  *0.57* | -0.014  *0.97* | 0.19  *0.60* | -0.013  *0.97* | **0.52 *0.005*** | - |  |  |  |
| LVMI  (g/ m^2.16^+0.09)^5^ | 0.033  *0.87* | -0.12  *0.55* | **-0.39**  ***0.045*** | 0.21  *0.57* | -0.16  *0.66* | 0.35  *0.33* | -0.13  *0.73* | 0.16  *0.4* | **0.76**  ***<0.001*** | - |  |  |
| LV mass Z-score^6^ | -0.042  *0.83* | -0.079  *0.69* | -0.35  *0.07* | 0.20  *0.58* | -0.096  *0.79* | 0.28  *0.43* | -0.06  *0.87* | 0.18  *0.4* | **0.78**  ***<0.001*** | **0.95**  **<0.001** | - |  |
| eGFR^7^ | -0.037  *0.84* | 0.16  *0.40* | 0.067  *0.73* | 0.39  *0.23* | -0.06  *0.86* | 0.17  *0.63* | -0.41  *0.21* | -0.37 *0.06* | -0.35  *0.07* | **-0.43**  **0.02** | **-0.41**  ***0.03*** | **-** |
| ^1^ Age at echocardiogram (years)  ^2^ Mean casual systolic and diastolic blood pressure (SBP, DBP) percentiles = mean percentile of casual SBP and DBP on day of echocardiogram and casual BPs recorded at up to 3 clinic visits within 6 months of echocardiogram  ^3^ Ambulatory blood pressure monitor indexes were calculated by dividing the mean SBP or DBP during wake or sleep by the upper limit of normal for age and sex, as defined by 2022 American Heart Association guidelines (for ≥ 13 years of age: 130/80 mmHg wake and 110/65 sleep; for <13 years of age: lower value of 95^th^ percentile or adolescent cut points) [27].  ^4^ left ventricular mass index indexed to height to the power of 2.7  ^5^ left ventricular mass index indexed to height to the power of 2.16 with a correction factor of 0.09)  ^6^ left ventricular mass (g), normalized to LV mass-for-height Z-score  ^7^ estimated glomerular filtration rate calculated using CKiD U25 creatinine-based equation [25], <https://ckid-gfrcalculator.shinyapps.io/eGFR/>  *Pearson’s correlation was used for all analyses except hypertension score, which used Spearman’s correlation. | | | | | | | | | | | | |

## Supplementary Table 2

**Linear regression analysis to assess relationships between the primary outcomes of interest, LVMI (indexed as either g/m^2.7^ or g/m^2.16^ + 0.09) and LV mass Z-score, and predictors including casual BP percentiles, ABPM indexes, hypertension score, age, and sex in patients with ARPKD**. β coefficients are scaled to show change in LVMI or LV mass Z-score per 5 mL/min/1.73m^2^ change in eGFR, per 0.1 change in ABPM indexes, and per 10 percentage point change in casual BP percentiles.

| **Outcomes**  **Predictors** | **LVMI in g/m^2.7^** | | **LVMI in g/(m^2.16^ + 0.09)** | | **LV mass Z-score** | |
| --- | --- | --- | --- | --- | --- | --- |
|  | β coefficient  (95% CI) | *P* | β coefficient  (95% CI) | *P* | β coefficient (95% CI) | *P* |
| **Univariate regression** | | | | | | |
| **Age** | **-2.39 (-4.20,-0.62)** | **0.01** | 0.10 (-1.06,1.25) | 0.9 | -0.012 (-0.12,0.10) | 0.83 |
| Mean casual SBP percentile^1^ | 2.54 (-1.50,6.59) | 0.21 | -1.00 (-4.37,2.37) | 0.6 | -0.062 (-0.38,0.26) | 0.69 |
| Mean casual DBP percentile^1^ | 1.16 (-1.60,3.92) | 0.40 | **-2.15 (-4.26, -0.05)** | **0.045** | -0.18 (-0.39,0.019) | 0.07 |
| ABPM Wake SBP index^2^ | 3.39 (-9.7,16.5) | 0.60 | 4.16 (-11.94, 20.27) | 0.6 | 0.31 (-0.92,1.55) | 0.58 |
| ABPM Wake DBP index^2^ | -0.16 (-9.44,9.13) | 0.97 | -2.25 (-13.49, 9.00) | 0.7 | -0.10 (-0.97,0.77) | 0.79 |
| ABPM Sleep SBP index^2^ | 3.18 (-10.4,16.8) | 0.57 | 7.23 (-8.70,23.15) | 0.3 | 0.45 (-0.80,1.70) | 0.43 |
| ABPM Sleep DBP index^2^ | -0.25 (-15.9,15.4) | 0.97 | -2.95 (-21.98, 16.08) | 0.7 | -0.11 (-1.58,1.36) | 0.87 |
| Hypertension score^3^ | **8.96 (2.95, 15.00)** | **0.005** | 2.27 (-3.39, 7.92) | 0.4 | 0.23 (-0.30, 0.77) | 0.4 |
| eGFR | -1.20 (-2.50,0.10) | 0.07 | **-0.84 (-1.56, -0.13)** | **0.02** | **-0.07 (-0.15,-0.007)** | **0.03** |
| **Adjusted for age & sex** | | | | | | |
| Mean casual SBP percentile^1^ | -3.54 (-9.32,2.23) | 0.22 | -2.37 (-7.11, 2.37) | 0.3 | -0.25 (-0.70,0.21) | 0.28 |
| Mean casual DBP percentile^1^ | -2.90 (-7.12,1.31) | 0.17 | -3.04 (-6.36, 0.28) | 0.07 | **-0.33 (-0.64,-0.01)** | **0.04** |
| ABPM Wake SBP index^2^ | 2.47 (-11,16) | 0.67 | 3.77 (-10.84, 18.38) | 0.6 | 0.25 (-0.96,1.46) | 0.63 |
| ABPM Wake DBP index^2^ | 1.45 (-9.53,12.40) | 0.76 | 1.86 (-10.20, 13.93) | 0.7 | 0.12 (-0.87,1.11) | 0.78 |
| ABPM Sleep SBP index^2^ | -3.00 (-20,14) | 0.68 | -1.60 (-20.47, 17.27) | 0.8 | -0.17 (-1.71,1.36) | 0.79 |
| ABPM Sleep DBP index^2^ | -4.12 (-22,14) | 0.59 | -5.09 (-24.48, 14.30) | 0.5 | -0.39 (-1.98,1.20) | 0.57 |
| Hypertension score^3^ | 5.86 (-1.50, 13.23) | 0.11 | 2.92 (-3.27, 9.11) | 0.3 | 0.23 (-0.37, 0.84) | 0.4 |
| eGFR | **-1.45 (-2.33,-0.17)** | **0.03** | **-0.82 (-1.44, -0.12)** | **0.01** | **-0.08 (-0.14,-0.01)** | **0.02** |
| **Adjusted for age, sex, and eGFR** | | | | | | |
| Mean casual SBP percentile^1^ | -1.95 (-6.97,3.07) | 0.43 | -1.25 (-5.60, 3.11) | 0.6 | -0.14 (-0.57,0.29) | 0.49 |
| Mean casual DBP percentile^1^ | -2.57 (-6.06,0.92) | 0.14 | -2.81 (-5.72, 0.10) | 0.058 | **-0.31 (-0.59,-0.024)** | **0.04** |
| ABPM Wake SBP index^2^ | 8.21 (-6.48,23) | 0.21 | 10.55 (-4.42, 25.50) | 0.1 | 0.73 (-0.65,2.10) | 0.23 |
| ABPM Wake DBP index^2^ | 1.66 (-9.86,13) | 0.73 | 2.08 (-10.57, 14.74) | 0.7 | 0.13 (-0.93,1.20) | 0.76 |
| ABPM Sleep SBP index^2^ | 1.90 (-21,25) | 0.84 | 4.73 (-19.73, 29.20) | 0.6 | 0.25 (-1.82,2.32) | 0.77 |
| ABPM Sleep DBP index^2^ | -5.75 (-24,13) | 0.46 | -6.89 (-26.91, 13.12) | 0.4 | -0.53 (-2.21,1.16) | 0.46 |
| Hypertension score^3^ | 1.40 (-5.86, 8.66) | 0.7 | -0.52 (-6.80, 5.75) | 0.9 | -0.09 (-0.71, 0.53) | 0.8 |
| **Adjusted for age, sex, and mean casual SBP and DBP** | | | | | | |
| eGFR | **-1.08 (-1.76,-0.41)** | **0.003** | **-0.79 (-1.35, -0.24)** | **0.007** | **-0.07 (-0.13,-0.02)** | **0.01** |
| **Adjusted for age, sex, and hypertension score** | | | | | | |
| eGFR | **-1.02 (-1.78, -0.27)** | **0.01** | **-0.79 (-1.44, -0.14)** | **0.02** | **-0.07 (-0.14, -0.01)** | **0.03** |
| ABPM, 24-hour ambulatory blood pressure monitor; DBP, diastolic blood pressure; eGFR, estimated glomerular filtration rate; LVH, left ventricular hypertrophy; LVMI, left ventricular mass index; LV mass Z-score, left ventricular mass normalized to LV mass-for-height Z-score; MAP, mean arterial pressure; SBP, systolic blood pressure.  ^1^ Mean casual SBP and DBP percentile = mean percentile of casual SBP and DBP on day of echocardiogram and casual BPs recorded at up to 3 clinic visits within 6 months of echocardiogram  ^2^ ABPM indexes were calculated by dividing the mean SBP or DBP during wake or sleep by the upper limit of normal for age and sex, as defined by 2022 American Heart Association guidelines (for ≥ 13 years of age: 130/80 mmHg wake and 110/65 sleep; for <13 years of age: lower value of 95^th^ percentile or adolescent cut points)  ^3^ Hypertension score = 1 point for each antihypertensive agent (based on medications at the visit closest to the echocardiogram) and 1 point for presence of hypertension, defined as overall mean casual SBP and/or DBP percentile >95th percentile. | | | | | | |

# Supplementary Figure legends

## Supplementary Figure 1

**Combinations of classes of blood pressure medications used in patients with ARPKD at the clinic visit closest to the echocardiogram**. ACE-I, Angiotensin converting enzyme inhibitor; ARB, angiotensin receptor blocker, BB, beta blocker; CCB, calcium channel blocker; CAAA, centrally acting alpha agonist.

## Supplementary Figure 2

**Relationship between left ventricular hypertrophy** (LVH, by Chinali et al. criteria [19]) **and BP in patients with ARPKD. A.** Box plot of casual SBP and DBP percentiles in patients with and without LVH; **B.** Box plot of ambulatory blood pressure monitor (ABPM) wake SBP and DBP indexes in patients with and without LVH; **C.** Box plot of ABPM sleep SBP and DBP indexes in patients with and without LVH.
