# Supplementary figures and images for: Determinants of left ventricular mass in children with autosomal recessive polycystic kidney disease
Source: J Nephrol. Author manuscript; Available in PMC 2025 Dec 22. (PMC12712119; doi:10.1007/s40620-025-02426-y)

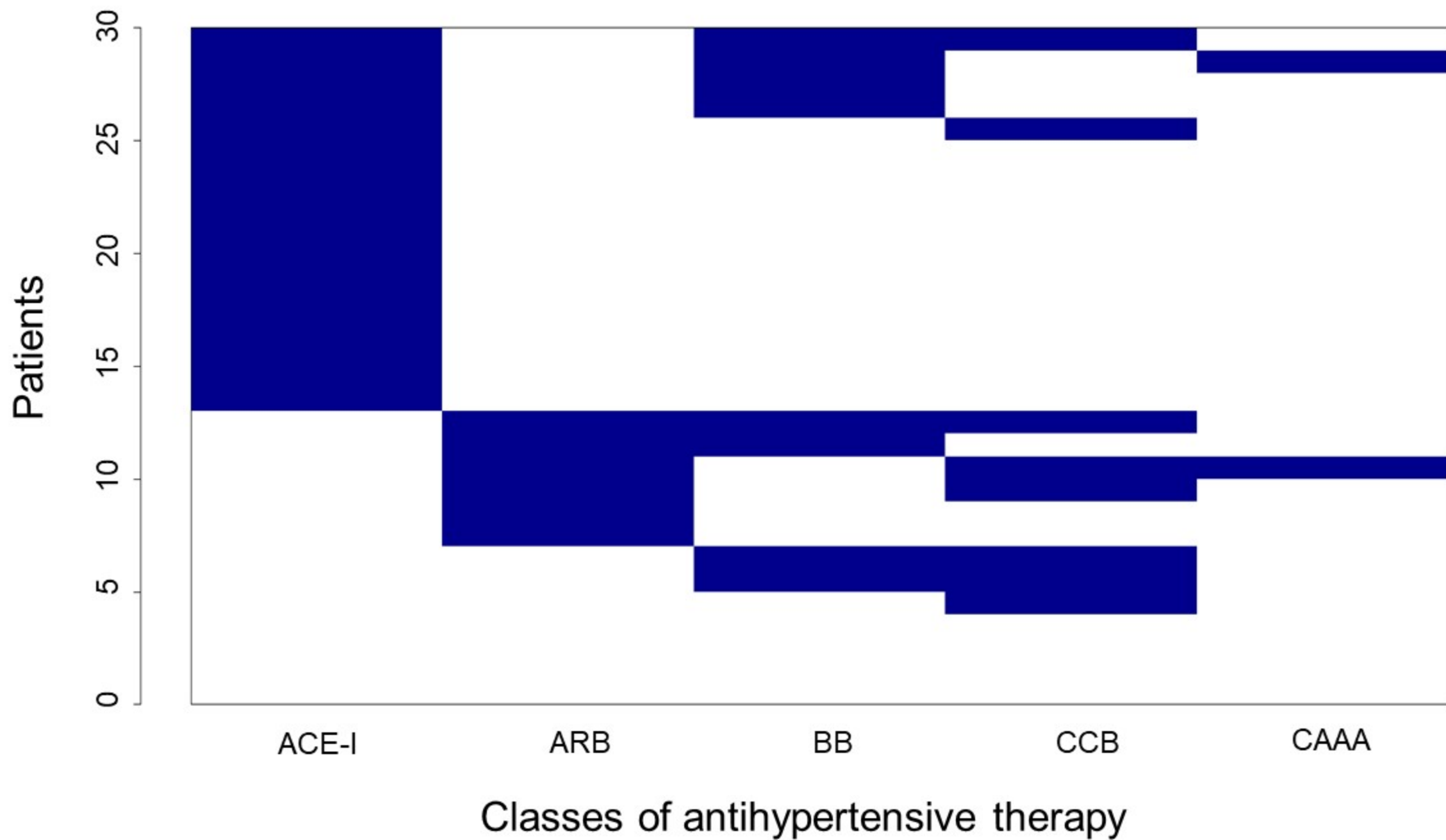

Supplement: Supplementary File 2 [file NIHMS2124282-supplement-Supplementary_File_2.pdf]

**A.**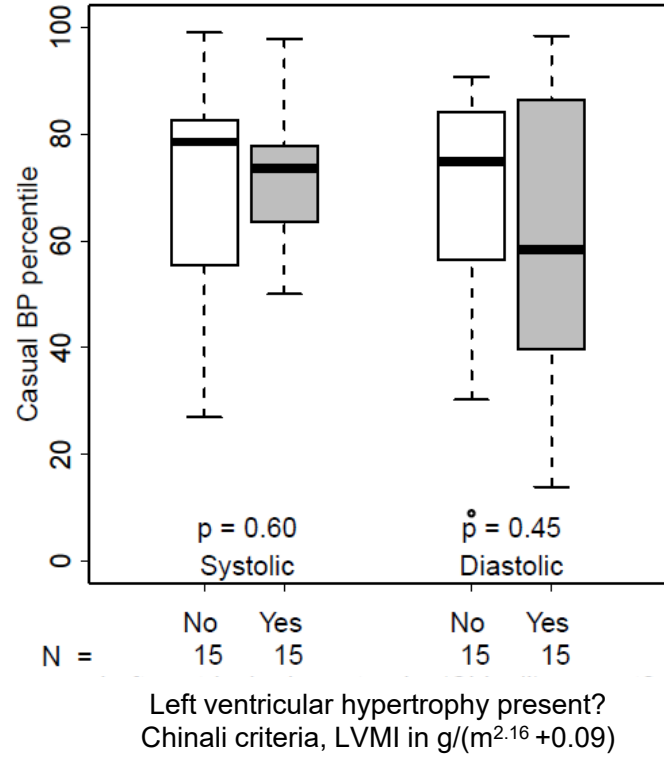**B.**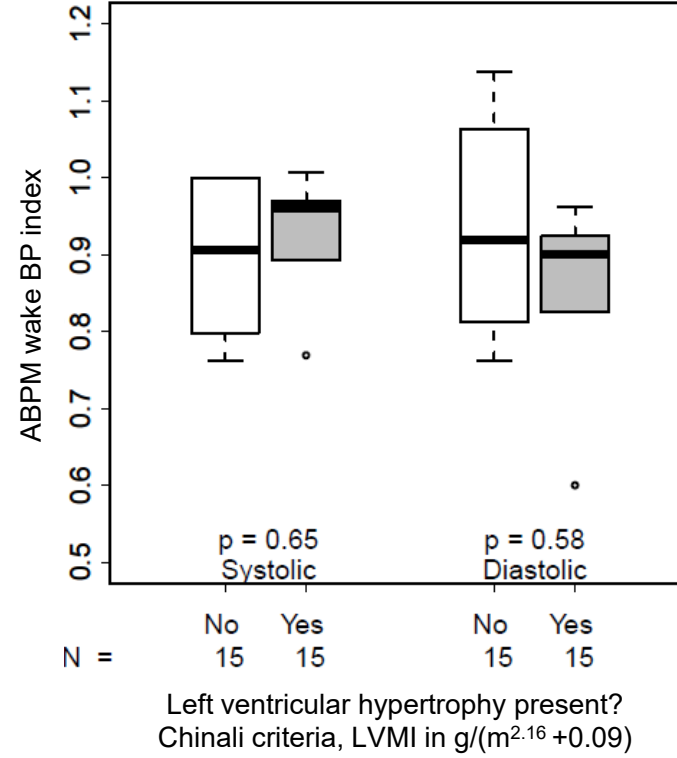**C.**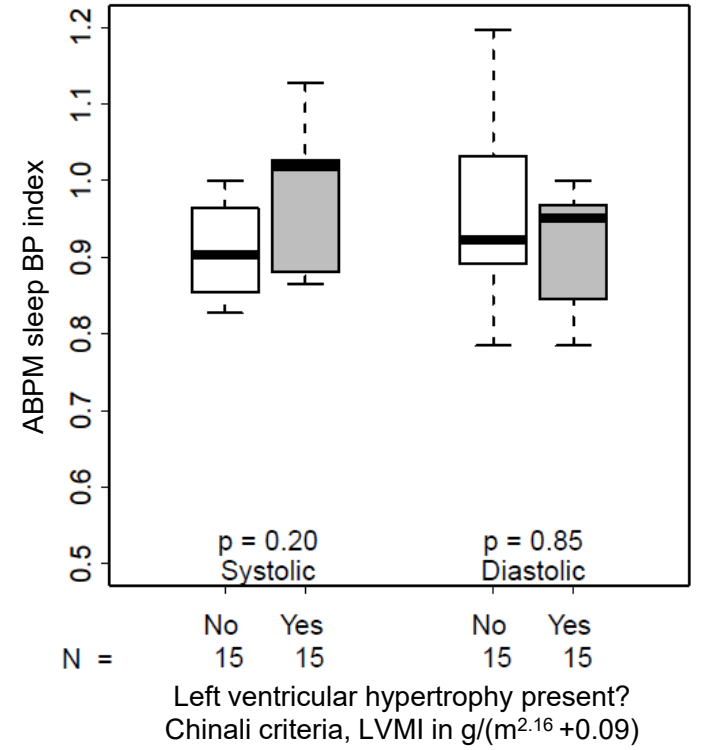

Supplement: Supplementary File 3 [file NIHMS2124282-supplement-Supplementary_File_3.pdf]
